# Single-Shot Measurement of Temporally-Dependent Polarization State of Femtosecond Pulses by Angle-Multiplexed Spectral-Spatial Interferometry

**DOI:** 10.1038/srep32839

**Published:** 2016-09-06

**Authors:** Ming-Wei Lin, Igor Jovanovic

**Affiliations:** 1 Institute of Nuclear Engineering and Science, National Tsing Hua University, Hsinchu 30013, Taiwan; 2 Department of Mechanical and Nuclear Engineering, The Pennsylvania State University, University Park, PA 16802, USA; 3 Department of Nuclear Engineering and Radiological Sciences, University of Michigan, Ann Arbor, MI 48109, USA

## Abstract

We demonstrate that temporally-dependent polarization states of ultrashort laser pulses can be reconstructed in a single shot by use of an angle-multiplexed spatial-spectral interferometry. This is achieved by introducing two orthogonally polarized reference pulses and interfering them with an arbitrarily polarized ultrafast pulse under measurement. A unique calibration procedure is developed for this technique which facilitates the subsequent polarization state measurements. The accuracy of several reconstructed polarization states is verified by comparison with that obtained from an analytic model that predicts the polarization state on the basis of its method of production. Laser pulses with mJ-level energies were characterized via this technique, including a time-dependent polarization state that can be used for polarization-gating of high-harmonic generation for production of attosecond pulses.

The common objective of ultrafast pulse characterization is to quantify the amplitude and phase of the electric field in spectral or, equivalently, in temporal domain. It may also be desirable or required to characterize the polarization state of the pulse^1^. It is well-known that the electric field associated with a laser pulse can be represented as a superposition of two component vectors that are orthogonally polarized. When an initially linearly polarized laser pulse propagates through a birefrigent dispersive medium such that its polarization is not parallel to one of the characteristic axes of the medium, its polarization can be converted into an elliptical state. In addition, the field envelopes of the two polarization components generally propagate at different group velocities in a birefringent media; consequently, a time-varying polarization state can be produced due to the temporal walk-off between them^2^.

Arbitrary manipulation of polarization and temporal profile of a laser pulse can be accomplished by use of a Michelson interferometer, which can be referred to as the global polarization state generator^3^. In a Michelson interferometer, two orthogonal polarization components of an input pulse are split and propagated along two independent paths. Arbitrary amplitude and phase modulation can be independently applied to each polarization component by use of modulators placed into the two interferometer beam paths. Finally, the two components are combined at the output of the interferometer to synthesize an output pulse. In the contemporary implementations, polarization shaping of a laser pulse is realized by means of active methods, such as by placing a spatial-light modulator (SLM) containing an array of programmable liquid crystal (LC) cells^4^,^5^,^6^ in the Fourier plane of a 4 *f* imaging system. By controlling the voltage applied to LC cells that introduce the spectral amplitude and phase modulation into their corresponding frequency components, polarization-shaped laser pulses with complex temporal variation throughout their pulse envelope can be produced.

One major application of femtosecond (fs) laser pulses with time-varying polarization is the production of attosecond pulses from high-order harmonic generation (HHG)^7^,^8^ driven by either few-cycle^9^ or by many-cycle laser pulses^10^. This technique is usually known as polarization-gating of HHG, in which a short time gate is realized by preparing a pulse that is linearly polarized only in the vicinity of the peak of a laser pulse. In the HHG process, the probability for an ionized electron to collide with its parent ion and recombine, whereby radiation is emitted, is very low for pulses that are not linearly polarized. Therefore, a short, transient, linearly polarized state occurring in a period between two elliptically polarized states can be used to define a sub-cycle gating window that allows the HHG radiation to be produced over an attosecond time scale. In this way, a relatively long femtosecond laser pulse can be used to drive HHG and generate isolated attosecond pulses. In comparison, an attosecond pulse train is produced when a conventional linear polarization is adopted^9^. Quantum control is another special application of polarization-shaped fs laser pulses that can enable the study of multiphoton and tunneling ionization processes^11^,^12^. In these applications, active pulse shaping was implemented to produce laser pulses with time-dependent polarization and interact with molecular beams, allowing the correlation between the ionization yield and the instantaneous polarization state to be observed. This in turn can lead to understanding of the underlying ionization mechanisms.

In all of those cases it is desirable to perform an accurate measurement of the polarization state of an ultrashort pulse. This capability rests upon the development of techniques that are able to conveniently measure the amplitudes and phases of the two polarization field components comprising the pulse. Among the common ultrashort pulse characterization techniques, Frequency-Resolved Optical Gating (FROG)^13^ and Spectral Phase Interferometry for Direct Electric-field Reconstruction (SPIDER)^14^ are now mature and have been widely adopted. However, they are designed to characterize laser pulses with linear polarization, either because of the use of a nonlinear process that requires linear polarization (for example, second-harmonic generation FROG or SPIDER) or by the underlying principle of the technique (for example, polarization-gating FROG).

In order to reconstruct the time-dependent polarization state of a laser pulse it is necessary to measure not only the amplitudes and phases of the two orthogonal field components, but also the relative phase between them. Time-resolved ellipsometry^15^,^16^ is one of the early techniques that was shown to be capable of measuring temporally-varying polarization states by measuring a sequence of all four Stokes parameters. This method could be time-consuming because the reference pulse delay has to be scanned sequentially over the entire pulse length in order to sample the signal field. Moreover, complicated configurations of waveplates are needed to make measurements that determine the Stokes parameters at each temporal delay position. Building upon spectral interferometry (SI), the POLarization Labeled Interference versus Wavelength of Only a Glint (POLLIWOG)^17^ technique has been successfully used for measuring polarization-shaped pulses. In POLLIWOG, the orthogonal components of the pulse under characterization (signal) are split and interfered with the corresponding components of a reference pulse in a spectrometer; in this way, two spectral interferograms can be obtained. The reference pulse has to be fully characterized by using a self-referenced technique, such as FROG or SPIDER, and the spectral amplitudes and phases of the signal components can be directly retrieved from the reconstruction of measured spectral fringes. Finally, the temporally varying field amplitudes and phases of reference components are obtained by Fourier transforming their spectral counterparts. A method that employs cross-phase modulation^18^ has also been adopted and improved to characterize the polarization evolution of a fs pulse. The pulse spectra before and after transmission through nonlinear Kerr media are measured and compared; an iterative algorithm is then used to retrieve the temporal amplitudes and phases of the two polarization components. Recently, the Tomographic Ultrafast Retrieval of Transverse Light E-fields (TURTLE)^19^,^20^,^21^,^22^ was demonstrated and used for characterization of polarization-shaped pulses^6^. In TURTLE, field components of a signal pulse are sampled by setting the orientation of a polarizer at three angles. Two of the measurements are taken with orthogonal field components with polarization adjusted so that they pass through a single polarizer, while the third sample is obtained by rotating the polarizer to an intermediate position. Those three linearly-polarized field projections are characterized separately (using FROG or SPIDER, for example), and their powers are also measured independently. The complete amplitudes and phases that determines the polarization state of the signal pulse can then be obtained by solving an unique analytical equation that describes the TURTLE measurement.

SI and spatial-spectral interferometry (SSI) techniques are sensitive and fast, making them attractive for characterizing the polarization states of ultrafast laser pulses. POLLIWOG is one variant of SI, in which the spectral amplitudes and phases of the polarized field components can be obtained by processing an interferogram generated without the use of a nonlinear process. However, the spectral resolution is sacrificed in the SI measurements due to a large time delay between the interfering pulses required to generate the spectral fringe pattern in collinear geometry. On the other hand, the SSI^23^ is performed by crossing the signal and reference pulses in the image plane with zero delay to directly record the spectral amplitude and phase differences between them. Therefore, SSI is capable of a higher spectral resolution than SI and has been applied as a powerful method that can accurately measure the material dispersion, angular dispersion, and carrier-envelope phase drift^24^,^25^. In a version of SSI called Spatially Encoded Arrangement for Temporal Analysis by Dispersing a Pair Of Light E-fields (SEA TADPOLE)^26^,^27^, a Fourier transform-based algorithm is developed to facilitate the extraction of the field amplitude and phase of a pulse from the SSI interferogram. A further extension of SSI is the use of an angularly multiplexed geometric arrangement in which two reference pulses are introduced to overlap with the signal pulse at different crossing angles^28^. Since the two reference pulses interfere separately with the two orthogonal polarization components of signal pulse, the generated SSI interferogram contains fringes at two spatial frequencies that can be separated by Fourier filtering and processed separately to obtain their field amplitudes and phases. This angle-multiplexed SSI technique has been demonstrated and used to characterize the relative spectral phase and the spatially variable polarization state of a radially polarized pulse^28^. It should be noted that interferometric techniques require a nontrivial effort to align the signal and reference pulses and to maintain good phase stability, such that a high-quality interferogram can be produced. In addition, if the absolute amplitudes and phases of the signal components are needed, each of the reference pulses have to be fully characterized using another self-referenced technique.

The goal of this work is to explore the capability of the angle-multiplexed SSI technique to measure temporally dependent polarization states of ultrafast laser pulses prepared using a combination of a birefringent quartz plate and a zero-order waveplate. A calibration procedure is developed that makes use of a linearly-polarized pulse independently characterized using the SPIDER technique, from which the spectral phases and dispersions of the two reference pulses can be determined. The calibrated phases of reference pulses are subsequently used to extract the spectral phases of the orthogonally polarized signal pulse components. Knowledge of the spectral amplitudes and phases of the signal field components enables the retrieval of their temporal counterparts and, consequently, the reconstruction the temporally-dependent polarization state of an ultrafast pulse. While the SSI is known for its high sensitivity (below a picojoule), the technique can also be used to measure more energetic pulses, such as those needed to drive HHG, after appropriate attenuation. We demonstrate the characterization of elliptically polarized pulses and compare the results with the prediction from an analytical model. We show that the angle-multiplexed SSI is capable of resolving the variations in the amplitudes and phases of the signal pulse polarization components in good agreement with an analytical model. To demonstrate the capability of the technique, we reconstruct the temporal evolution of a polarization state resembling that used in polarization gating of HHG for generation of attosecond pulses.

## Results

An experimental setup was constructed based on the SSI approach (see Supplementary Note 1). Following the system calibration, signal pulses with various polarization states were generated by adjusting the angles of a waveplate (*θ*_*p*_) and a quartz plate (*θ*_*z*_), as described in Supplementary Note 3. The signal pulses can be parametrized by their two orthogonal field components, 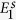 and 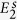. The measured interferograms were reconstructed using the method described in Supplementary Note 4. Here we succinctly present the principle of system calibration developed for angle-multiplexed SSI and the results obtained with elliptically polarized state and a time-varying polarization state typical for polarization gating of HHG.

### System calibration using linearly polarized state

Accurate calibration is essential for success of an interferometric measurement^14^,^26^,^28^ using the constructed setup shown in Supplementary Note 1. As discussed in earlier work^28^, the calibration process in spatial-spectral SSI includes setting the delay between the two reference pulses and the signal pulse to zero, determining the exact spectral phases of the two reference pulses, and compensating for any differences in the spectrally dependent transmission of the orthogonal polarization components of a signal pulse. The previously demonstrated calibration method identifies the spectral phase difference of the two references pulse, from which the spectral phase difference *δϕ* (*ω*) of the signal field components and the relevant spectral elliptical parameters can be obtained^28^. However, this method does not provide the individual phases of the reference pulses 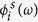, which are necessary to obtain the spectral phases 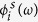 of the two signal field components. Since the temporal amplitudes of signal field components 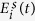 are necessary for determining the temporal elliptical parameters, as evident from Eqs (S1) and (S2), the spectral amplitudes 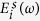 and phases 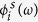 of the signal field components have to be extracted from the measurement. Therefore, a major requirement is to first calibrate the exact spectral dispersion of the two reference pulses 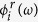.

**Figure 1:**
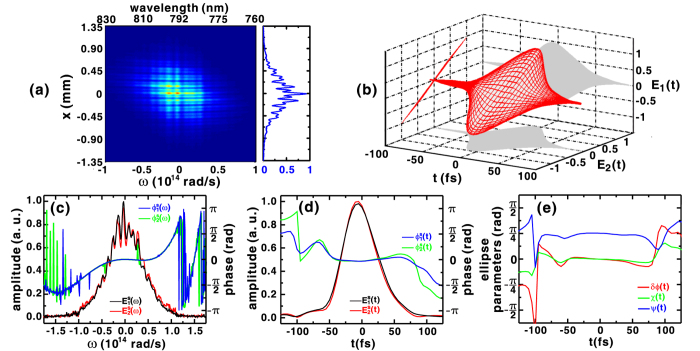
(**a**) Experimental SSI interferogram of the 45° oriented, linearly polarized signal pulse used in the calibration procedure, while the right side shows the line-out profile taken at *λ*_0_ = 792 nm. (**b**) Temporal evolution of the polarization state of signal pulse that is reconstructed from the amplitudes and phases of the field components illustrated in the (**c**) spectral and (**d**) temporal domains. (**e**) Temporal evolution of the retrieved polarization ellipse parameters *δϕ(t*), *χ(t*), and *ψ(t*) that describe the temporal polarization state shown in (**b**).

To accomplish the calibration in a simple and reliable manner, a procedure involving linearly polarized pulses oriented at 45° is developed and used in this work. Such pulse suitable for calibration can be produced by setting the quartz plate and quarter-waveplate angles to *θ*_*z*_ = −45° and *θ*_*p*_ = 45°, respectively, as illustrated in Supplementary Note 3. As a result, the orthogonal field components of the linearly polarized signal pulse have identical spectral amplitudes and phases, making them suitable for use in calibration. Figure 1(a) shows an interferogram measured for the purpose of system calibration, along with the vertical profile taken around *λ*_0_ = 792 nm that reveals the superposition of the two spatial frequencies. Here, the delays of the reference pulses are adjusted to make the fringes horizontal, so that their zero-delay positions with respect to the signal pulse can be determined.

In the second step of the calibration process the spectral phases 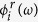 of the two reference pulses are determined. From the measured SSI interferogram, the phase difference *δϕ*_*i*_(*ω*) = 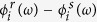 between the corresponding polarization components of the signal and reference pulses can be extracted and used to retrieve the absolute phases 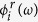 of reference pulses when 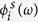 of the signal pulse is known. In this work, the SPIDER technique^29^ was used to measure the spectral phase 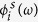 of the signal pulse. The spectral phase *ϕ* (*ω*) can be expressed as


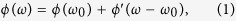


in terms of *ϕ(ω*_0_), the absolute phase at a central frequency *ω*_0_ and *ϕ*′(*ω* − *ω*_0_), a function that describes high-order dispersion. Hence, the phase difference *δϕ*_*i*_(*ω*) between the signal pulse and each reference pulse is


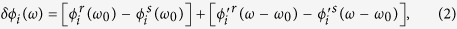


which contains the absolute phase difference


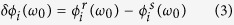


and the difference between the functions describing their high-order spectral dispersion:


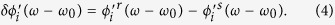


By using SPIDER to characterize this 45° oriented, linearly polarized signal pulse, a calibration phase dispersion 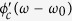 can be applied to both orthogonal components of the signal pulse 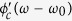 = 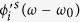, since they are in phase. Note that in the SPIDER measurements the calibrated absolute phase at frequency *ω*_0_ is always *ϕ*_*c*_(*ω*_0_) = 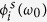 = 0. After each phase difference *δϕ*_*i*_(*ω*) is obtained, the phase 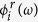 of the corresponding reference pulse can be determined from Eqs (3) and (4) as


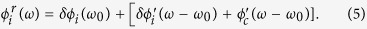


Following the calibration process, the phases of the two reference pulses 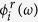 are used in the phase retrieval algorithm. Therefore, the phases of signal pulse components 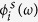 = 
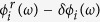
 can be extracted in the subsequent measurements when the phase differences *δϕ*_*i*_(*ω*) are updated.

Polarization-dependent spectral reflectivity functions of the wedge and the grating dominate the distinct spectral response functions for each orthogonal component of the signal pulse. The used retrieval algorithm accounts for the spectral transmission functions when reconstructing the spectral field amplitudes 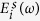. Transmission functions *f*_*i*_(*ω*) of each polarization component are determined by comparing the uncorrected field amplitudes retrieved from a measured interferogram with the one independently measured with a spectrometer. The transmission functions are used to accurately retrieved the field amplitudes 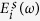 needed to reconstruct the polarization state.

After the calibration procedure is complete, the spectral phases 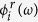 of the reference pulses and the spectral transmission functions *f*_*i*_(*ω*) of the signal pulse are determined and applied in the algorithm to process subsequent measurements. Figure 1(b) shows the temporal field reconstructed from the interferogram in Fig. 1(a), confirming the linear polarization state of the signal pulse used for calibration. The corresponding field amplitudes and phases are shown in Fig. 1(c), in which the spectral phase functions are in good agreement with the dispersion *ϕ*_*c*_(*ω* − *ω*_0_) measured by the SPIDER technique. By taking the inverse Fourier transform of the spectra, the corresponding temporal field amplitudes and phases can be retrieved, as shown in Fig. 1(d), which determine the overall time-dependent field evolution shown in Fig. 1(b). The temporal phase difference *δϕ(t*) shown in Fig. 1(e) is nearly constant at zero in the temporal range −50 fs < *t* < 50 fs, which means that the majority of the field components 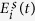 are in phase. Moreover, the corresponding ellipticity angle 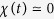 confirms the linear polarization state of signal pulse. Thus, the reconstructed orientation angle 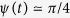 (or 45°) within the −50 fs < *t* < 50 fs is in good agreement with expectation.

### Measurement of elliptically polarized state

**Figure 2:**
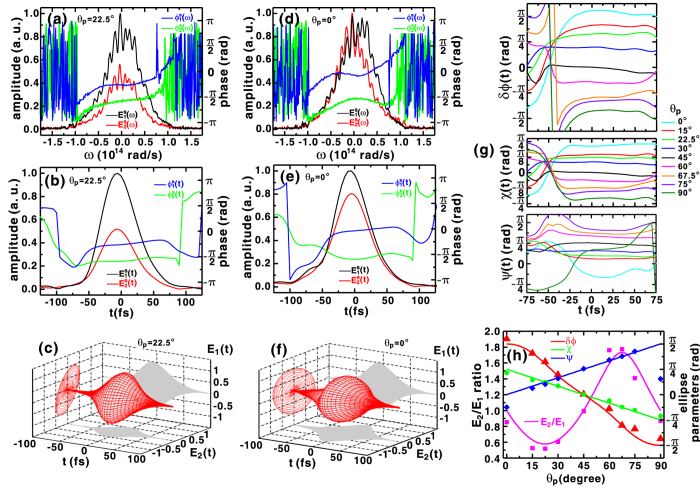
When the quarter-waveplate is rotated to *θ*_*p*_ = 22.5°, the retrieved (**a**) spectral and (**b**) temporal amplitudes and phases of the polarization components determine the temporal polarization state shown in (**c**). (**d–f**) illustrate the corresponding amplitudes, phases, and the temporal polarization state when the quarter-waveplate is rotated to *θ*_*p*_ = 45°. (**g**) Comparison of the temporal evolution of retrieved polarization ellipse parameters *δϕ(t*), *χ(t*), and *ψ(t*), corresponding to various angles of the quarter-waveplate *θ*_*p*_. (**h**) Dependence of the retrieved polarization ellipse parameters *δϕ*(0), *χ*(0), and *ψ*(0) at time *t* = 0 on the set angle of the quarter-waveplate *θ*_*p*_. All of the data were obtained with the quartz plate set at a fixed angle *θ*_*z*_ = −45°.

Signal pulses are assigned various ellipticities to validate the ability to reconstruct their polarization states from the measured field amplitudes and relative spectral phase shifts 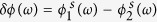 between the two orthogonal field components of the signal, where *ω* is the angular frequency. While keeping the quartz plate angle fixed at *θ*_*z*_ = −45°, successive rotations of the quarter-waveplate (see Supplementary Note 3) generated a range of phase shifts *δϕ* (*ω*). Hence, the relative temporal phase shift *δϕ(t*) and the polarization state varied accordingly. Figure 2(a) shows the measured field amplitudes and spectral phases when the quarter-waveplate was rotated to *θ*_*p*_ = 22.5°. Compared to the results shown in Fig. 1(c), in which a linearly polarized pulse was considered, the reduced amplitude *E*_2_(*ω*) and the disparity between phases 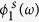 and 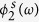 characterize the production of an elliptically polarized pulse with *θ*_*p*_ = 22.5°. The corresponding temporal fields and phases shown in Fig. 2(b) show the reduction of the amplitude ratio of *E*_2_(*t*) to *E*_1_(*t*) (the peak ratio 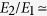 0.52), and a retrieved phase difference 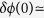 0.324*π* at *t* = 0. The temporal field profile of an elliptically polarized pulse is reconstructed as shown in Fig. 2(c), in which an ellipticity angle 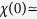 0.122*π* and an orientation angle 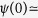 0.1*π* is obtained.

The effect of the transmission through a quarter-waveplate on the two polarization field components can be described by the Jones matrix formalism:


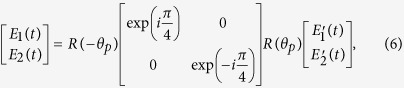


in which the output fields *E*_1,2_(*t*) are determined by the input fields 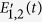, and the matrix


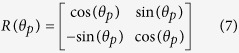


accounts for the coordinate transformation between the laboratory *y* axis and the optic axis at a given angle *θ*_*p*_. For a linearly polarized input pulse oriented at 45°, 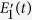 = 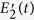 = 1, and *θ*_*p*_ = 22.5° yields the expected field ratio *E*_2_/*E*_1_ = 0.577, phase difference *δϕ* = 0.304*π*, ellipticity angle *χ* = 0.125*π*, and orientation angle *ψ* = 0.125*π*. Therefore, the retrieved experimental results shown in Fig. 2(b) are a good agreement with theoretical predictions.

Figure 2(d) shows the measured spectral amplitudes and phases when the quarter-waveplate was rotated to *θ*_*p*_ = 0. Compared to the results shown in Fig. 2(a), the magnitude of 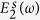 increased to a level similar to 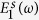, but a greater phase difference was produced. Figure 2(e) shows the retrieved temporal amplitudes and phases, which indicate a peak ratio 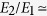 0.85 and a phase difference 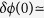 0.55*π* at *t* = 0. Those values are close to the calculated peak ratio *E*_2_/*E*_1_ = 1 and phase difference 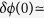 0.5*π* when setting *θ*_*p*_ = 0 in Eq. (6). From the results shown in Fig. 2(e), a temporal field profile resembling a circularly polarized pulse is reconstructed, as shown in Fig. 2(f). At *t* = 0, the retrieved ellipticity angle is 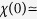 0.214*π*, in reasonable agreement with the calculated value of *χ*(0) = 0.25*π* estimated by Eq. (6). However, the retrieved orientation angle 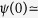 −0.124*π* deviates from the ideal result of *ψ* = 0. Considering the definition of the retrieved orientation angle *ψ(t*) in Eq. (S2), the deviation results primarily from the departure of the retrieved phase difference 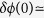 0.55*π* from the calculated *δϕ*(0) = 0.5*π*. The quarter-waveplate was successively rotated from *θ*_*p*_ = 0 to 90°. Figure 2(g) summarizes the retrieved relative temporal phase shifts and the polarization ellipse parameters in the temporal interval −75 fs < *t* < 75 fs, where the field has relatively high amplitude. Figure 2(h) compares the reconstructed field component parameters at *t* = 0 with the values predicted using Eq. (6). The measurements indicate that *δϕ*(0) varies linearly in the range ± *π*/2, *χ*(0) varies linearly in the range ± *π*/4, and *ψ*_0_ varies between 0 and *π*/2. Therefore, we conclude that the measured elliptical polarization parameters shown in Fig. 2(g,h) are in good agreement with their expected values, except for the retrieved orientation angle *ψ*(0) that corresponds to circularly polarized pulses in the case *θ*_*p*_ = 0 and 90°. Because the function in Eq. (S2) used to calculate the orientation angle *ψ(t*) (see Supplementary Note 2) diverges when the peak ratio *E*_2_/*E*_1_ and phase shift *δϕ(t*) approach unity and ± *π*/2, respectively, it can be understood that this definition makes it challenging to obtain the correct orientation angle when characterizing a circularly polarized pulse.

**Figure 3:**
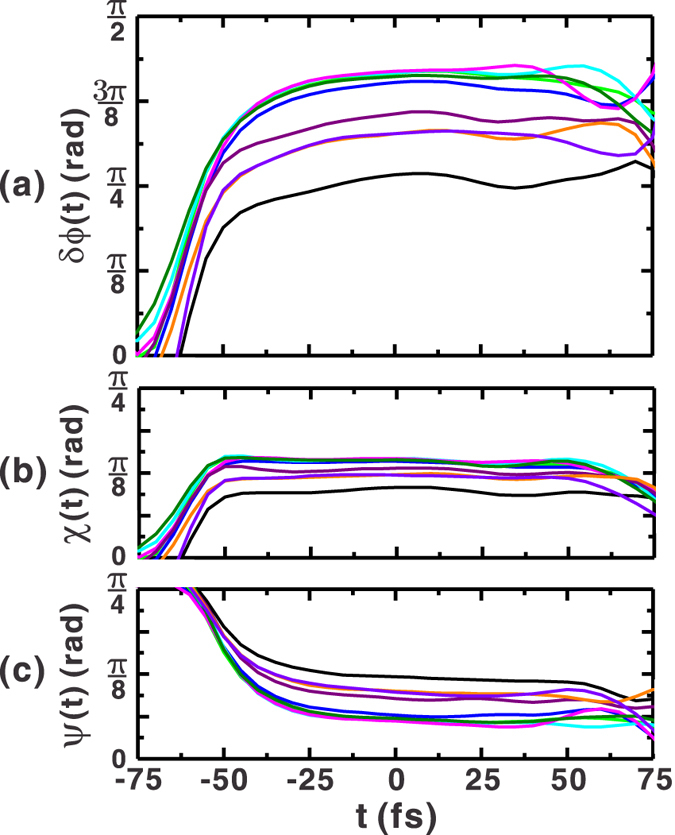
A series of polarization ellipse parameters retrieved from nine consecutive measurements when *θ*_*p*_ = 22.5° and *θ*_*z*_ = −45° were set for the quarter-waveplate and the quartz plate, respectively.

As discussed previously^26^,^28^, a small change of the optical path lengths for the signal and two reference pulses can be manifested as fast jitter and a slow drift in the spectral phase. This phase error also leads to the uncertainty of the retrieved temporal phase difference and the ellipse parameters. The jitter and drift of measured phase may originate from the mechanical vibrations and refractive index changes due to the air currents and temperature fluctuations during the period over which the measurement is performed. In this work, all measurements were performed within five minutes after each system calibration in order to reduce the effect of slow phase drift. Therefore, only the fast jitter contributes significantly to the variation of the retrieved phase. Nine successive measurements were taken with quarter waveplate angle set to *θ*_*p*_ = 22.5° over one minute. Figure 3(a) shows the retrieved temporal phase difference, while the corresponding ellipticity and orientation angles are shown in Fig. 3(b,c), respectively. The uncertainties of these retrieved pulse parameters can be estimated from the mean-value subtracted root mean square (RMS) variation at *t* = 0, which are in order: 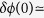 0.15 rad, 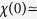 0.04 rad, and 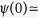 0.07 rad. The energy stability of the laser can also induce errors in the retrieved polarization ellipse parameters because the reference pulse spectra 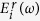 are measured independently prior to the measurement. Consequently, the energy fluctuation of the laser pulses causes the fluctuation of reconstructed field amplitudes. In addition, errors in the retrieved ellipse parameters can be amplified when the output energy stability degrades because of their dependence on the field amplitude, which is evident from Eqs (S1) and (S2). In our experiment, the stability of the laser pulse energy was ≈1%. Therefore, the the variations in the retrieved field parameters originated primarily from the fast phase jitter.

### Measurement of the temporally varying polarization state

The ability to characterize a laser pulse with a temporally varying polarization state was demonstrated by measuring pulses that exhibit a group delay between the field envelopes of their two orthogonal polarization components. By setting the quartz plate angle to *θ*_*z*_ = 0, the *E*_1_(*t*) field component, polarized in *x* direction, experiences the refractive index *n*_*o*_, while the *E*_2_(*t*) field component, polarized in *y* direction, experiences the refractive index *n*_*e*_. At the output of the plate the field envelopes of *E*_1_(*t*) and *E*_2_(*t*) are temporally separated by


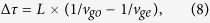


where *L* is the thickness of the quartz plate and *v*_*go*_ and *v*_*ge*_ are the group velocities of the two field envelopes that can be calculated from


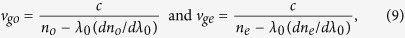


where *c* is the speed of light in vacuum. At *λ*_0_ = 792 nm in quartz, *n*_*o*_ = 1.539 and *n*_*e*_ = 1.547^30^, which gives 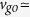 0.643*c* and 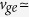 0.639*c*. Applying Eq. (8) with a thickness of *L* = 1.15 mm, the envelope of 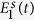 leads that of 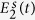 by a temporal separation 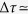 36.6 fs. Passage through the subsequent quarter-waveplate can be used to further change the polarization state of the signal pulse. Note that the group velocity dispersion caused by the second zero-order waveplate can be neglected because it introduces only sub-cycle phase changes.

**Figure 4:**
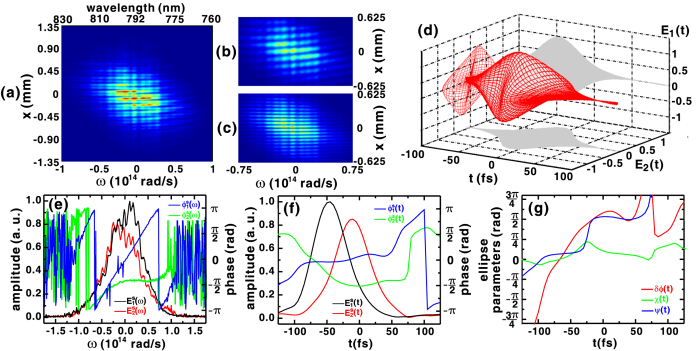
(**a**) SSI interferogram measured with the quartz plate and the quarter-waveplate set to *θ*_*z*_ = *θ*_*p*_ = 0 and when only the (**b**) vertically or (**c**) horizontally polarized reference pulse were introduced. (**d**) Temporal evolution of the polarization state of the signal pulse reconstructed from the amplitude and phase of the field components shown in (**e**) spectral and (**f**) temporal domain. (**g**) Temporal evolution of the retrieved polarization ellipse parameters that describe the temporal polarization state shown in (**d**).

When the quarter-waveplate is set to *θ*_*p*_ = 0, the *E*_1_(*t*) and *E*_2_(*t*) fields retain their linear polarization state, since their orientations are parallel to the slow- and fast-axis of the waveplate, respectively. The corresponding interferogram is shown in Fig. 4(a). Figure 4(b) shows the interferogram recorded when only the vertically (*x*) polarized reference pulse was permitted to interfere with the vertically polarized component of signal pulse, while Fig. 4(c) shows the corresponding result between the horizontally (*y*) polarized reference pulse and the horizontally polarized signal pulse component. The fringes shown in Fig. 4(b) are tilted when compared to the those shown in Fig. 4(c). As reported earlier^23^,^25^,^28^, a non-zero group delay between the signal and reference pulses causes the tilt of interference fringes, in this case along the horizontal direction. Since the zero-delay position for the reference pulses was determined in the calibration procedure when the signal pulse was transmitted through the quartz plate with a group velocity *v*_*ge*_, the change of group velocity of 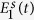 to *v*_*go*_ produces a fringe tilt. In contrast, fringes shown in Fig. 4(c) are nearly horizontal, which confirms the group velocity of *v*_*ge*_ for 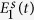.

Figure 4(d) shows the temporal profile of the signal pulse polarization components reconstructed from the spectral amplitudes and phases in Fig. 4(e), while their temporal counterparts are shown in Fig. 4(f). The polarization state gradually changes from linear in *x* to nearly circular at the front of the pulse and then evolves to a linear state in *y* at the back of pulse. The sloped spectral phase 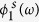 shown in Fig. 4(e) manifests itself as the significant temporal advance of 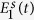, as seen in Fig. 4(g). The retrieved separation between the peaks of 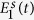 and 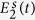 of Δ*τ* ≈ 35 fs is in good agreement with the value calculated via Eqs (8) and (9). As shown in Fig. 4(g), the retrieved ellipticity angle is near zero at the front and rear part of the pulse, and reaches a peak of 0.22*π* at *t* = −25 fs. The temporal orientation angle *ψ(t*) also changes from 0 near the front of the pulse to approximately *π*/2 at its trailing edge, confirming the temporal pulse profile shown in Fig. 4(d).

**Figure 5:**
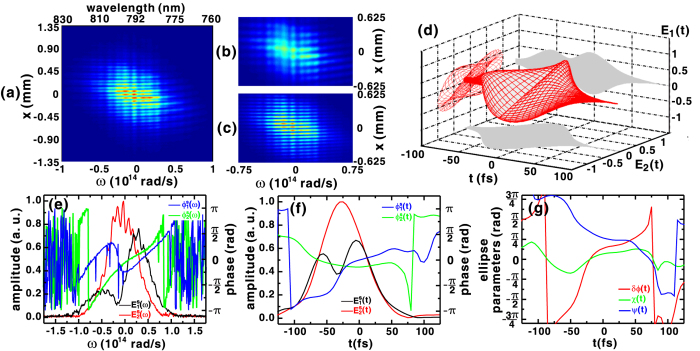
(**a**) Measured SSI interferogram when the quartz plate and the quarter-waveplate were set to *θ*_*z*_ = 0 and *θ*_*p*_ = −45°, respectively. Also shown are the measured SSI interferograms when only the (**b**) vertically and (**c**) horizontally polarized reference pulse was introduced. (**d**) Temporal evolution of the polarization state of the pulse reconstructed from the amplitude and phase of the field components shown in the (**e**) spectral and (**f**) temporal domain. (**g**) Temporal evolution of the retrieved polarization ellipse parameters that describe the temporal polarization state shown in (**d**).

Figure 5(a) shows the interferogram produced when the quarter-wave plate was rotated to *θ*_*p*_ = −45°. In this situation, the signal pulse is similar to that used in polarization gating of HHG^9^,^10^,^31^. The polarization state of a pulse produced in this manner is elliptical or circular at the leading and trailing side of the pulse, but becomes linear in the middle of the pulse. Figure 5(b) shows the interferogram measured when the signal pulse was interfered with the vertically polarized reference pulse. A discontinuity in the fringe pattern between the low- and high-frequency components indicates a near-*π* phase jump introduced in field 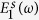 of the signal pulse at that quarter-waveplate setting. This type of discontinuity in the spectral phase is similar to that previously measured by SSI when a phase retarder was placed in a 4*f* pulse shaper^23^. In contrast, the fringe pattern shown in Fig. 5(c) indicates that the spectral phase of 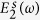 is continuous.

Figure 5(d) shows the reconstructed temporal profile of the signal pulse for which the polarization state changes from elliptical to linear and back to elliptical. The corresponding spectral amplitudes and phases are shown in Fig. 5(e), resulting in the temporal amplitudes and phases shown in Fig. 5(f). The *π*-phase jump near the frequency *ω* = −0.17 × 10^14^ rad/s (or the wavelength *λ* = 798 nm) seen in Fig. 5(b) produces a corresponding depression of the spectral amplitude in 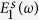 shown in Fig. 5(e) and the appearance of a dip in the middle of the retrieved temporal amplitude 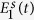 in Fig. 5(f). More importantly, the temporal phases 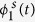 and 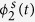 intersect with each other at *t* ≈ −20 fs, as shown in Fig. 5(f), which means that the phase difference is *δϕ(t*) = 0 and a transient linear polarization state was created. The ellipticity angle *χ(t*) shown in Fig. 5(g) indicates that the signal pulse is elliptically polarized at its leading and trailing edge.

## Discussion

A capability to measure temporally dependent polarization states of ultrafast laser pulses was experimentally demonstrated using the angle-multiplexed SSI technique. When compared to POLLIWOG^17^ and TURTLE^19^, this technique embodies the high speed and sensitivity of SEA TADPOLE^26^, while providing a high spectral resolution. By introducing two orthogonally polarized reference pulses and interfering them with the corresponding polarization components of the signal pulse, the angle-multiplexed SSI is capable of characterizing the polarization state of a laser pulse in a single-shot measurement. This unique property helps to reduce errors that can be introduced by splitting the signal pulse and performing two interferometric measurements, as done in POLLIWOG. Instead of performing separate measurements to measure the exact spectral phase of the two reference pulses, a different calibration procedure has been developed that makes use of linearly polarized pulses oriented at 45°. If the signal pulse used for calibration has been fully characterized by a self-referencing technique such as FROG^13^ or SPIDER^14^, the absolute spectral phase of two reference pulses, including their relative phase difference at the center wavelength, can be obtained and used for subsequent measurement. The technique is limited by the slow phase drift and shot-to-shot phase jitter^28^. However, it is shown experimentally in this work that the relative phase differences of elliptically polarized pulses can be retrieved with accuracy that compares favorably to analytical predictions. We demonstrated that mJ-level laser pulses can also be characterized via the angle-multiplexed SSI technique, including the class of pulses with time-dependent polarization characteristic for polarization-gating of HHG. It is expected that this technique will be useful for measurement of laser pulses with complex variations of the polarization state, such as those created by active pulse shaping devices^32^. The high spectral resolution offered by SSI can resolve the small manipulations applied to the spectral phase of the signal pulse components, which are needed to successfully reconstruct the complicated variation of the polarization state of a laser pulse.

## Methods

### Experimental setup

An experimental setup was constructed to facilitate production and measurement of various test polarization states. A detailed description and schematic of the experimental setup is provided in Supplementary Note 1. The polarization states are quantified in a standard fashion by their ellipticity angle and orientation angle, which is described in Supplementary Note 2. A two-component polarization converter comprised of a birefringent quartz plate and a quarter-waveplate was used to generate a range of polarization states for testing, including a linear, elliptical, and complex temporally varying polarization state resembling that used to gate HHG production for HHG generation. More details on this setup and production method are provided in Supplementary Note 3.

### Retrieval of temporally dependent polarization state

The method relies on the use of spatial Fourier filtering for extraction of two angularly multiplexed interferograms, from which the two orthogonal reference and two orthogonal signal components can be retrieved. This is described in more detail in Supplementary Note 4.

## Additional Information

**How to cite this article**: Lin, M.-W. and Jovanovic, I. Single-Shot Measurement of Temporally-Dependent Polarization State of Femtosecond Pulses by Angle-Multiplexed Spectral-Spatial Interferometry. *Sci. Rep.* **6**, 32839; doi: 10.1038/srep32839 (2016).

## Supplementary Material

Supplementary Information
