# Supplementary material for: Single-Shot Measurement of Temporally-Dependent Polarization State of Femtosecond Pulses by Angle-Multiplexed Spectral-Spatial Interferometry
Source: Sci Rep. 2016 Sep 6;6:32839. doi: 10.1038/srep32839 (PMC5011769; doi:10.1038/srep32839)
Supplement: Supplementary Information [file srep32839-s1.pdf]

Single-Shot Measurement of Temporally-Dependent Polarization State of Femtosecond Pulses by Angle-Multiplexed Spectral-Spatial Interferometry

Supplementary Information

Ming-Wei Lin[1,3] and Igor Jovanovic[2,3]

[1]Institute of Nuclear Engineering and Science, National Tsing Hua University, Hsinchu 30013, Taiwan
[2]Department of Nuclear Engineering and Radiological Sciences,
University of Michigan, Ann Arbor, MI 48109, USA
[3]Department of Mechanical and Nuclear Engineering,
The Pennsylvania State University, University Park, PA 16802, USA

Supplementary Note 1: Experimental setup

Figure S1 illustrates the complete layout of the experimental setup. The spectrometer is composed of a spherical mirror (SM_2), a grating (GR), a cylindrical mirror (CM) and a 12-bit CCD camera. The spectrometer allows spatially resolved measurements in one dimension (along the spectrometer slit). A linearly polarized laser pulse was provided by a conventional Ti:sapphire chirped-pulse

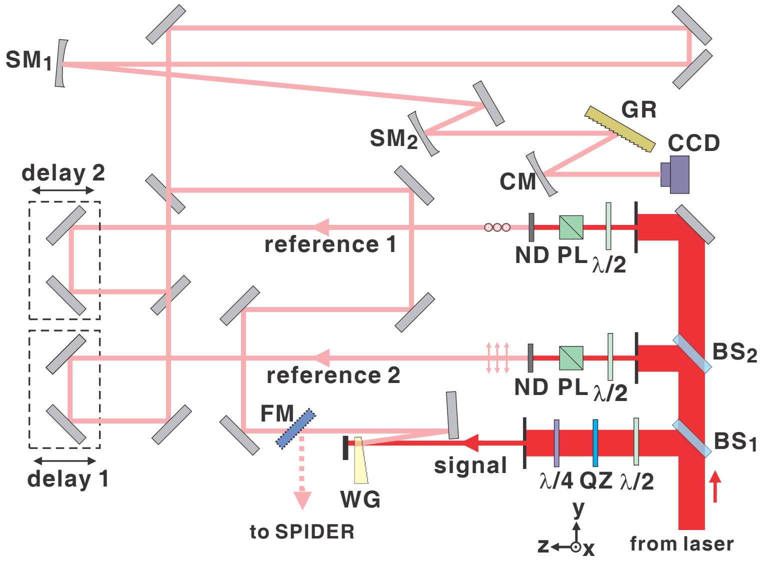

Figure S1: Experimental layout developed for characterization of a laser pulse with a temporally varying polarization state using the angle-multiplexed spatial-spectral interferometry technique. Details are given in the Methods.

amplification system, and had a full-width at half-maximum (FWHM) bandwidth of ~28 nm at the center wavelength $\lambda_0 = 792$ nm. The signal and two reference pulses were prepared using two

beamsplitters with a clear aperture size of 10 mm and a fixed input pulse energy of 2 mJ. The 1-mJ pulse reflected from the first beamsplitter (BS_1) served as the signal pulse, while the two reference pulses were produced by the second beamsplitter (BS_2). Various polarization states can be generated by adjusting the rotation angles of the quartz plate (QZ) and the zero-order half- and quarter-waveplates ($\lambda/2$ and $\lambda/4$). Since the spherical mirror SM_1 focuses the pulses before their arrival at the spectrometer slit, the signal pulse has to be attenuated in order to avoid the ionization of air at the focus. Therefore, an iris with a 3-mm aperture was used to sample the signal pulse in the center of the beam. By reflecting the pulse from the wedge WG, the sampled pulse was attenuated to 5 µJ for measurements. The polarization of the two reference pulses was set to linear, in the x (vertical) and y (horizontal) direction, and the two pairs of half-waveplates and polarizers (PL) were used to improve the polarization purity of the reference pulses. Irises and neutral density filters (ND) were introduced into the reference pulse path as well, so that their aperture sizes and pulse energies were approximately equal to those of the sampled signal pulse. Separate delay stages for reference pulses were positioned so they could be temporally overlapped with the signal pulse. Those three pulses were aligned in the same vertical plane and incident onto the spectrometer slit at different angles; a spatial-spectral interferogram was produced when they were spatially and temporally overlapped. The half-crossing angles between the signal and the two reference pulses were $\theta_1 \sim 0.16°$ and $\theta_2 \sim 0.32°$, respectively. A flip mirror (FM) could temporarily direct the signal pulse to a Spectral Phase Interferometry for Direct Electric-field Reconstruction (SPIDER) setup [S1] for measuring its spectral phase as a part of system calibration.

Supplementary Note 2: Definition of ellipticity angle and orientation angle

Propagating along the z direction, the signal pulse comprising two orthogonal polarization components can be described in terms of the temporal field amplitudes $E^s_{1,2}(t)$, laser frequency ω_0, and temporal phases $\phi^s_{1,2}(t)$ of its field components: $\mathbf{E}^s(t) = E^s_1(t)\cos(\omega_0 t + \phi^s_1(t))\hat{x} + E^s_2(t)\cos(\omega_0 t + \phi^s_2(t))\hat{y}$. The amplitudes $E^s_{1,2}(t)$ and the relative phase difference $\delta\phi(t) = \phi^s_1(t) - \phi^s_2(t)$ can be used to describe the complete polarization state of the signal pulse, which can be represented by a polarization ellipse, as illustrated in Fig. S2. The associated ellipticity angle $\chi(t)$ and orientation angle $\psi(t)$ can be calculated as

$$\sin 2\chi(t) = \frac{2E^s_1(t)E^s_2(t)}{E^s_1(t)^2 + E^s_2(t)^2}\sin\delta\phi(t), \qquad -\frac{\pi}{4} \le \chi(t) \le \frac{\pi}{4}, \tag{S1}$$

$$\tan 2\psi(t) = \frac{2E^s_1(t)E^s_2(t)}{E^s_1(t)^2 - E^s_2(t)^2}\cos\delta\phi(t), \qquad 0 \le \psi(t) \le \pi. \tag{S2}$$

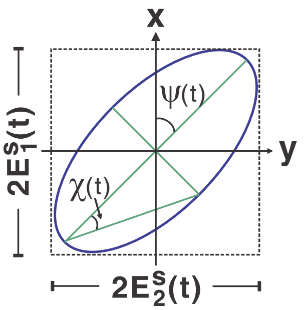

Figure S2: The polarization ellipse and the definitions for the ellipticity angle $\chi(t)$ and orientation angle $\psi(t)$.

Supplementary Note 3: Generation of temporally dependent polarization state

Pulses with well-defined time-dependent ellipticity can be prepared by transmitting a linearly polarized pulse through a polarization converter comprising a birefringent quartz plate and a zero-order quarter-waveplate, as illustrated in Fig. S3 [S2, S3]. In our experiments, an additional zero-order half-waveplate was used in the signal beam path to rotate the orientation of the linearly polarized signal pulse to a fixed orientation angle ψ=45° before the polarization converter. Subsequently, a 1.15-mm thick, multi-order waveplate was used to perform the function of a quartz plate. By varying the θ_z and θ_p angles, defined as the orientations of the optical axes of the quartz plate and the quarter-waveplate, respectively, with respect to the x direction, a range of polarization states can be generated. For example, a linearly polarized signal pulse is generated by setting θ_z=$\theta_p = \pm 45°$. If $\theta_z = 90°$ and $\theta_p = 45°$ are used, the polarization state changes from elliptical to linear and back to elliptical, which is similar resembles that used in polarization gating of HHG [S2, S3].

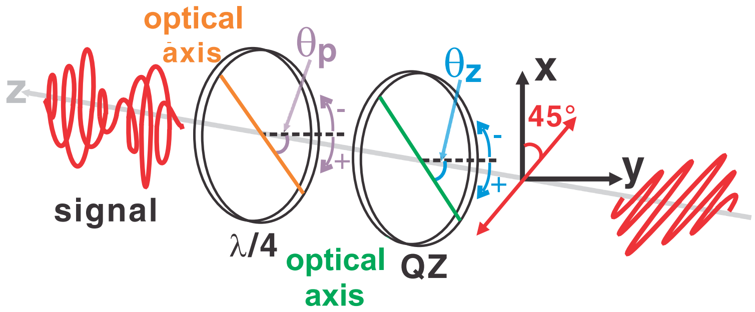

Figure S3: Illustration of a polarization converter comprised by a quartz plate (QZ) and a quarter-waveplate ($\lambda/4$) used for adjusting the polarization state of the signal pulse.

Supplementary Note 4: Retrieval of temporally dependent polarization state

As shown in earlier work with the angle-multiplexed SSI, the relative spectral phases and amplitude spectra of the two orthogonal field components of the pulse can be simultaneously retrieved from a single measurement [S4]. In the spectral domain, the signal pulse can be decomposed as $\mathbf{E}^s(\omega)$=$E_1^s(\omega)\exp[i(\vec{k}_1^s \cdot \vec{z} + \phi_1^s)]\hat{x}$ + $E_2^s(\omega)\exp[i(\vec{k}_2^s \cdot \vec{z} + \phi_2^s)]\hat{y}$, where $E(\omega)_{1,2}^s$ are the spectral amplitudes of the orthogonal polarization components, $\vec{k}^s$ is the wave number, $\hat{x}$ and $\hat{y}$ are the unit vectors in directions x and y, respectively, and $\phi_{1,2}$ are the spectral phases of the two polarization components. Two reference pulses, $E_1^r(\omega)\exp[i(\vec{k}_1^r \cdot \vec{z} + \phi_1^r)]$ and $E_2^r(\omega)\exp[i(\vec{k}_2^r \cdot \vec{z} + \phi_2^r)]$, polarized in the x and y directions, respectively, are temporally overlapped with the signal pulse on the spectrometer slit. The resulting interferogram $I(\omega, x)$ is given by $\left|\vec{E}_i^r + \vec{E}_i^s\right|^2$, or

$$I(\omega, x) = \sum_{i=1}^{2}[I_i^s(\omega) + I_i^r(\omega) + 2\sqrt{I_i^s(\omega)I_i^r(\omega)}\cos(2kx\sin\theta_i + \phi_i^s(\omega) - \phi_i^r(\omega))], \quad (S3)$$

where i=1, 2 represent the x and y directions, respectively, $I_i^s(\omega)$ and $I_i^r(\omega)$ are the spectral intensities, θ_i are the half-crossing angles between signal and reference pulses, and $\phi_i^s(\omega)$ and $\phi_i^r(\omega)$ are the spectral phases of signal and reference pulses. Since the reference pulses have different crossing angles ($\theta_{1,2}$), $I(\omega, x)$ contains interleaved fringes along the vertical dimension (x) at two spatial frequencies, which are inversely proportional to the two crossing angles. Figure S4 summarizes the 1-D Fourier-filtering algorithm that can retrieve the spectral amplitude and phase of the signal

components from a measured $I(\omega, x)$ trace [S4, S5]. Four sidebands can be identified in the $\omega - k_x$ domain, from which two neighboring sidebands are filtered and inverse-Fourier transformed back to the $\omega - x$ domain, so that each resulting product $|E_i^s E_i^r| \exp[i(\phi_i^s - \phi_i^r]$ contains the interference between a signal component and a corresponding reference pulse. By dividing these products by an independently measured $E_i^r(\omega)$, the amplitude $E_i^s(\omega)$ and phase difference $\delta\phi_i(\omega)$=$\phi_i^r(\omega) - \phi_i^s(\omega)$ can be extracted. As long as the phase $\phi_i^r(\omega)$ of a reference pulse has been determined, the phase of the pulse $\phi_i^s(\omega)$=$\phi_i^r(\omega) - \delta\phi_i(\omega)$ can be obtained. Using the retrieved spectral amplitudes $E_{1,2}^s(\omega)$ and phases $\phi_{1,2}^s(\omega)$ of the signal pulse, the corresponding temporal field components $E_{1,2}^s(t)$ and $\phi_{1,2}^s(t)$ can be calculated using the inverse Fourier transform. In addition, the temporal varying ellipticity of the signal pulse can be reconstructed using Eqs. (S1) and (S2).

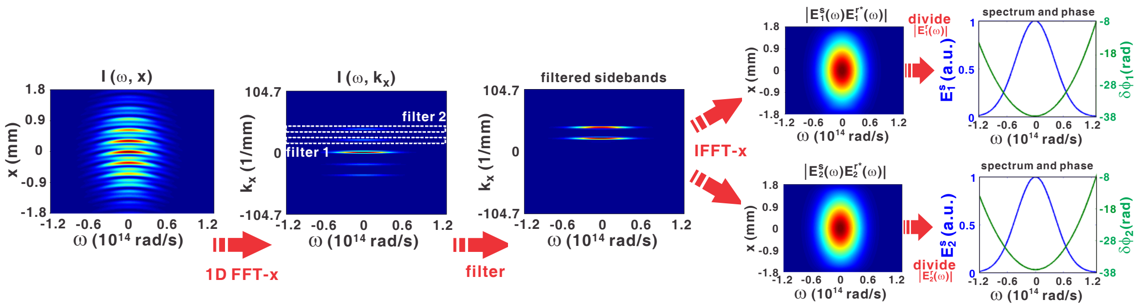

Figure S4: Flow diagram of the Fourier transform-based algorithm for retrieval of the relative spectral phases and amplitudes of the orthogonal field components of the measured pulse [S4].

Supplementary References

[S1] French, D., Dorrer, C., & Jovanovic, I. Two-beam SPIDER for dual-pulse single-shot characterization. *Opt. Lett.* **34,** 3415–3417 (2009).

[S2] Tcherbakoff, O., Mével, E., Descamps, D., Plumridge, J. & Constant, E. Time-gated high-order harmonic generation. *Phys. Rev. A* **68,** 043804 (2003).

[S3] Shan, B., Ghimire, S. & Chang, Z. Generation of the attosecond extreme untraviolet spuercontinuum by a polarization gating. *J. Mod. Opt.* **52,** 277–283 (2005).

[S4] Rakhman, A., Lin, M.-W. & Jovanovic, I. Angle-multiplexed spatial-spectral interferometry for simultaneous measurement of spectral phase and polarization state. *Opt. Express* **21,** 26896–26907 (2013).

[S5] Bowlan, P., Gabolde, P., Shreenath, A., McGresham, K. & Trebino, R. Crossed-beam spectral interferometry: a simple, high-spectral-resolution method for completely characterizing complex ultrashort pulses in real time. *Opt. Express* **14,** 11892–11900 (2006).
